# Supplementary material for: Recurrent DNA break clusters drive replication-stress-induced copy number variants and genome diversification
Source: Nat Commun. 2026 Apr 20;17:3627. doi: 10.1038/s41467-026-71790-5 (PMC13096501; doi:10.1038/s41467-026-71790-5)
Supplement: Supplementary file 1 — Supplementary Information [file 41467_2026_71790_MOESM1_ESM.pdf]

**Recurrent DNA Break Clusters Drive Replication-Stress-Induced Copy Number Variants and Genome Diversification**

Lorenzo Corazzi[1,2*], Alex Ing[1*], Eva Benito[3], Marco Raffaele Cosenza[3], Patrick Hasenfeld[3], Thomas Weber[3], Anna Marx[1,2], Vivien S. Ionasz[1], Nathan Trausch[1,2], Sarah Benedetto[4], Giulia Di Muzio[1,2], Boyu Ding[1,5], Jana Berlanda[1,2], Marco Giaisi[1], Nina Claudino[4], Thomas Höfer[4], Jan O. Korbel[3,6], Pei-Chi Wei[1,2,**]

Affiliations

1. Brain mosaicism and tumorigenesis laboratory, German Cancer Research Center, 69120 Heidelberg, Germany
2. Faculty of Bioscience, Ruprecht-Karl-University of Heidelberg, 69120 Heidelberg, Germany
3. European Molecular Biology Laboratory (EMBL), Genome Biology Unit, 69117 Heidelberg, Germany
4. Theoretical system biology laboratory, German Cancer Research Center, 69120 Heidelberg, Germany
5. Faculty of Medicine, Ruprecht-Karl-University of Heidelberg, 69120 Heidelberg, Germany
6. Bridging Research Division on Mechanisms of Genomic Variation and Data Science, German Cancer Research Center, 69120 Heidelberg, Germany

* These authors contributed equally

** Correspondence: Pei-Chi Wei (p.wei@dkfz-heidelberg.de)

## SUPPLEMENTARY INFORMATION


| Content | Pages |
|---|---|
| Supplementary Figures and Legends | 2-12 |

## Supplementary Figure 1

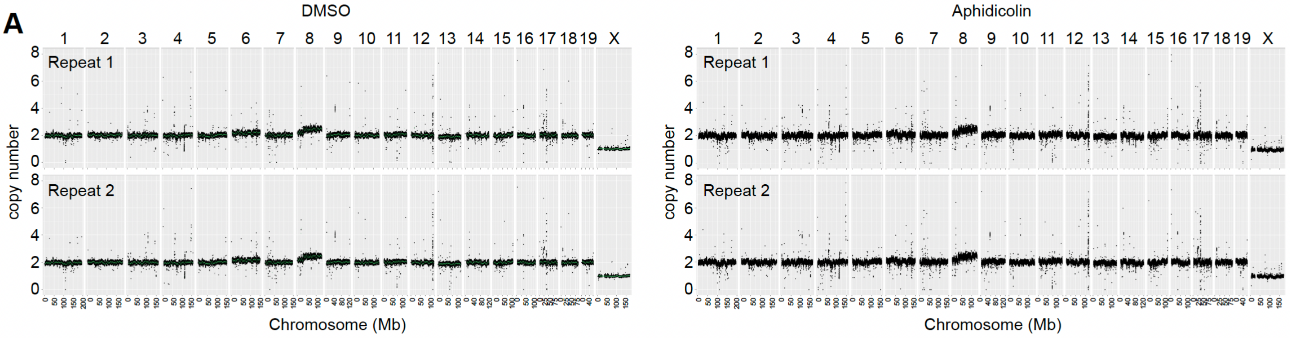


**Supplementary Figure 1. XRCC4/p53-deficient neural progenitor cell's genomes were mostly diploid.** The figure presents four ploidy plots for all autosomes and the X chromosome in samples treated with either the solvent control (DMSO) or aphidicolin. The Y-axis represents copy number, and the X-axis represents chromosome length in megabases.

## Supplementary Figure 2

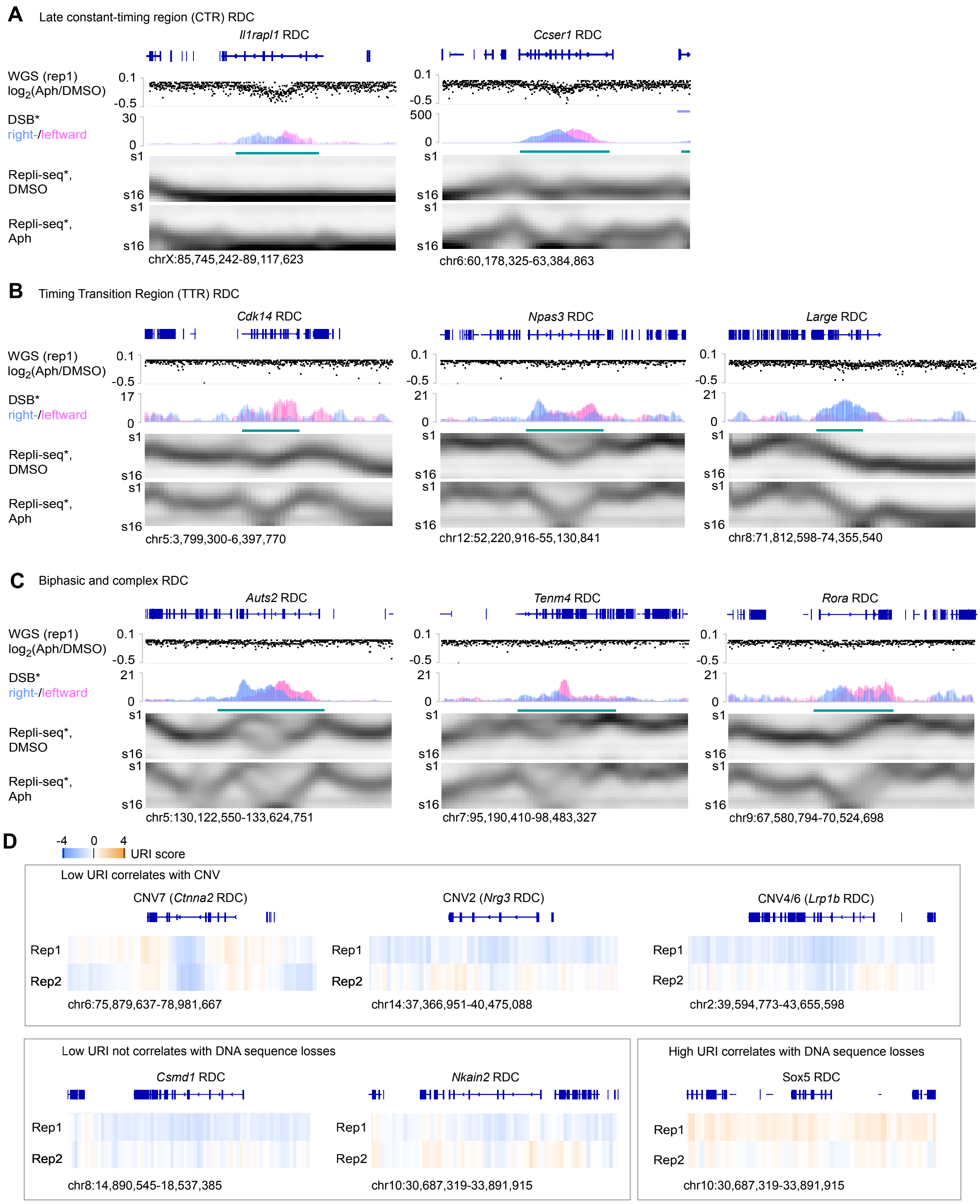


**Supplementary Figure 2. Aphidicolin-induced heterozygous deletions arise at a subset of late-replicating constant-timing RDC, continued. (A)** Two RDC locations exhibited significant DNA sequence losses near the Delly2 cutoff. **(B)** Three RDCs are located at the timing transition region (TTR). **(C)** Three RDCs are located at the biphasic replicating region. Tracks in Panel A-C are as in Fig. 2A. **(D)** Underreplication index (URI) and whole-genome sequencing $log_2$(APH/DMSO) for six RDC-containing genomic regions. Four loci - *Ctnna2*, *Lrp1b*, *Nrg3*, and *Csmd1* - exhibited low URI values and significant DNA sequence loss. At the *Sox5* locus, despite a high URI, a notable loss of DNA sequences was still

observed. In contrast, the *Nkain2* locus displayed a low URI without corresponding DNA sequence loss. The heatmap scale is shown above.

## Supplementary Figure 3

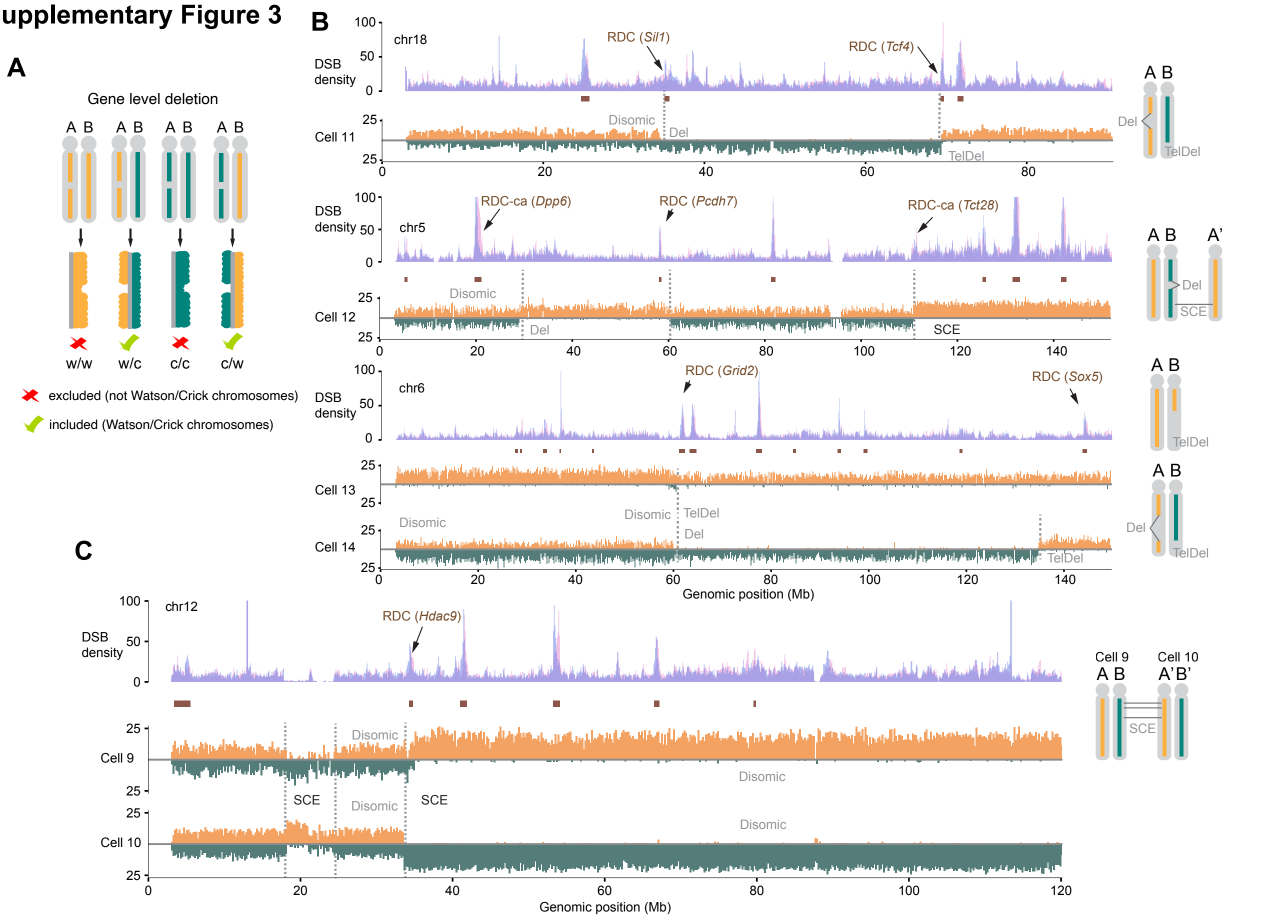


**Supplementary Figure 3. Strand-seq Detection Strategy for Gene-Level Deletions and Analysis of Inheritance Patterns (A)** Schematic illustrating the strategy for detecting gene-level deletions using Strand-seq. Detection is only possible in cells or chromosomal regions where both Watson and Crick orientations are inherited. In homozygous states (e.g., Watson-Watson), deletions on the missing strand cannot be distinguished. **(B)** Representative Strand-seq tracks displaying various structural alterations in single cells. RDC-ca: RDC candidates defined from prior investigation[18]. **(C)** Strand-seq profiles of chromosome 12 for two daughter cells (Cell 9 and Cell 10) derived from the same parental mitosis. The plots show mirrored Watson and Crick strand patterns, confirming a reciprocal Sister Chromatid Exchange (SCE) that occurred in the parental cell. Both daughters remain disomic across the exchange region, indicating the SCE event itself was balanced and did not result in copy number loss.

## Supplementary Figure 4

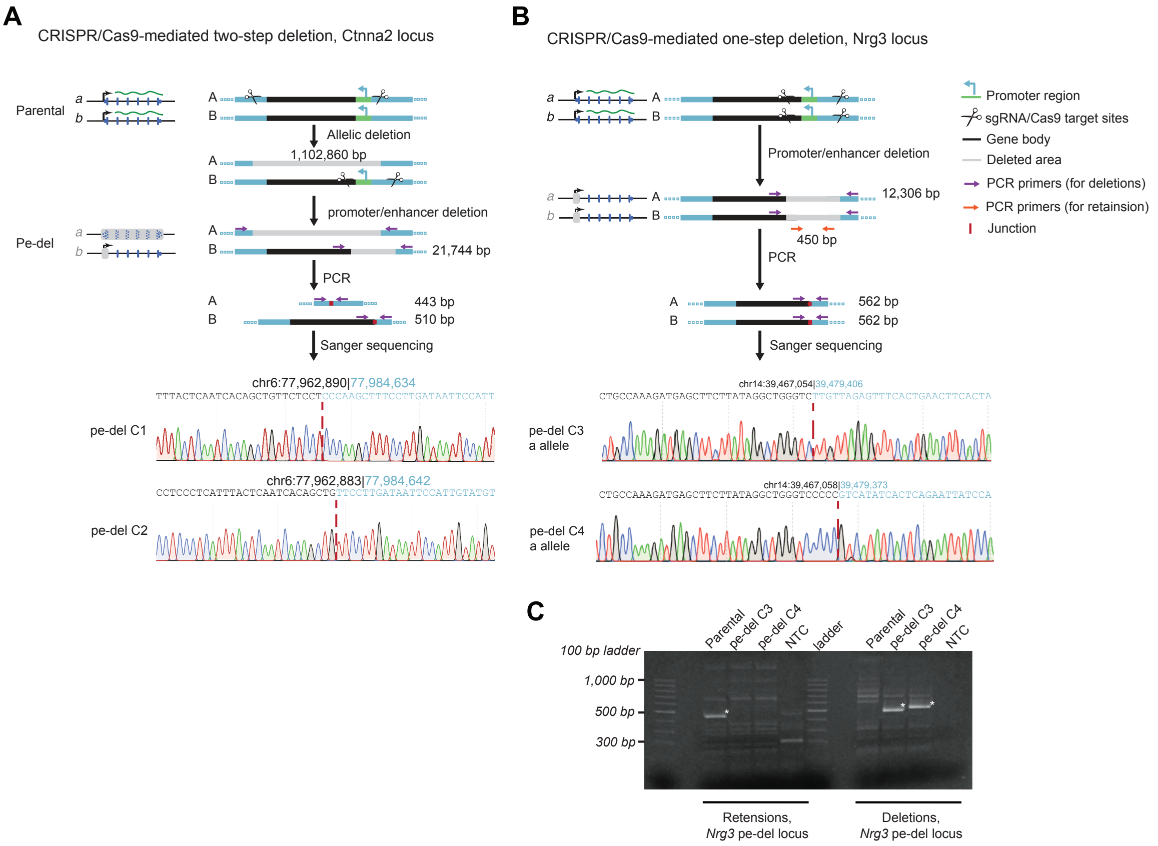


**Supplementary Figure 4. CRISPR/Cas9-mediated deletion strategies and validation for *Ctnna2* and *Nrg3* loci. (A)** Two-step deletion strategy at the *Ctnna2* locus. Schematic of CRISPR/Cas9-mediated allelic deletion (step 1, 1,102,860 bp) followed by promoter/enhancer deletion (step 2, 21,744 bp). Parental configuration (top) and promoter/enhancer deletion (Pe-del) configuration (middle) are shown, with sgRNA/Cas9 target sites indicated by scissors and deleted regions in black. PCR primers for deletion (purple) and retention (orange) assays are indicated, with expected amplicon sizes noted. Sanger sequencing traces from independent *Ctnna2* Pe-del clones (C1 and C2) show the junction sequences, with target coordinates (mm10) labeled and the deletion breakpoints indicated by red dashed lines. **(B)** One-step deletion strategy at the *Nrg3* locus. Schematic of CRISPR/Cas9-mediated promoter/enhancer deletion (12,306 bp) in a single step. PCR primers and expected amplicon sizes are indicated. Sanger sequencing traces from *Nrg3* Pe-del clones (C3 and C4) show deletion junctions, with mm10 coordinates and breakpoints indicated as in (A). **(C)** PCR genotyping of *Nrg3* Pe-del clones. Agarose gel electrophoresis showing amplicons for retention (left panel) and deletion (right panel) assays in parental, Pe-del clones (C3 and C4), and none-template controls (NTC). Expected band sizes correspond to schematics in (B). Asterisks indicate the expected PCR products. As Sanger sequencing detected only one junction in either the C3 or C4 clones, these results indicate that both clones harbor a deletion on either the a or b allele extending beyond the regions targeted by the deletion-specific primers. An uncropped image was uploaded as Source Data.

## Supplementary Figure 5

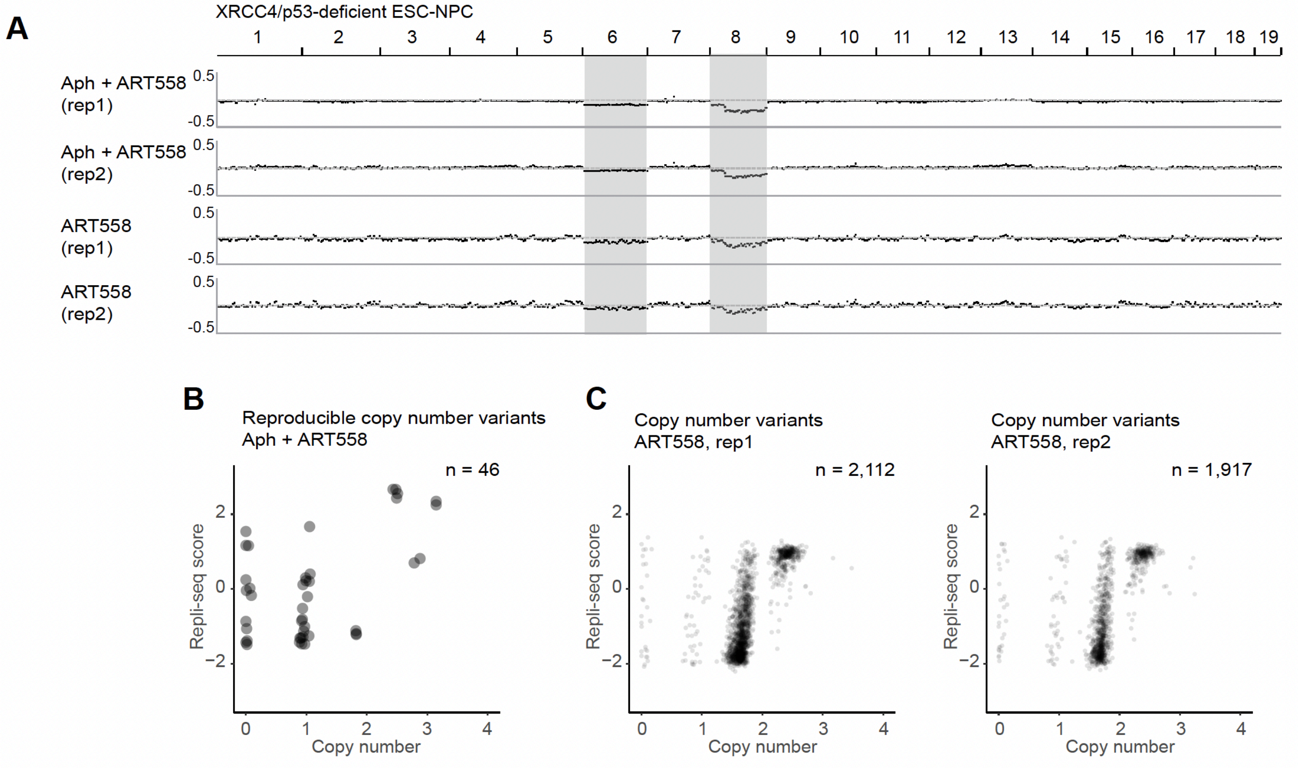


**Supplementary Fig. 5. Polθ inhibition promotes subclonal selection and alters DNA replication in XRCC4/p53-deficient neural progenitor cells.** (A) Whole-genome sequencing coverage ratios for all autosomes (chromosomes 1–19) across treatment conditions and experimental replicates. Binned sequencing coverage under each condition was normalized to the untreated control (DMSO) and plotted as $\log_2$ ratios (y-axis). Two significant and reproducible copy-number losses were observed on chromosomes 6 and 8. The corresponding CNVs are annotated in Supplementary Data 1. **(B)** Scatter plot showing the relationship between copy number and replication timing for 46 reproducible CNVs identified outside chromosomes 6 and 8 in XRCC4/p53-deficient neural progenitor cells treated with aphidicolin and ART558. **(C)** Scatter plots showing copy number versus replication timing for significant CNVs identified in two independent experimental replicates under ART558-only treatment. The number of CNVs included in each plot is indicated. Source data are provided as a Source Data file.

**Supplementary Figure 6**

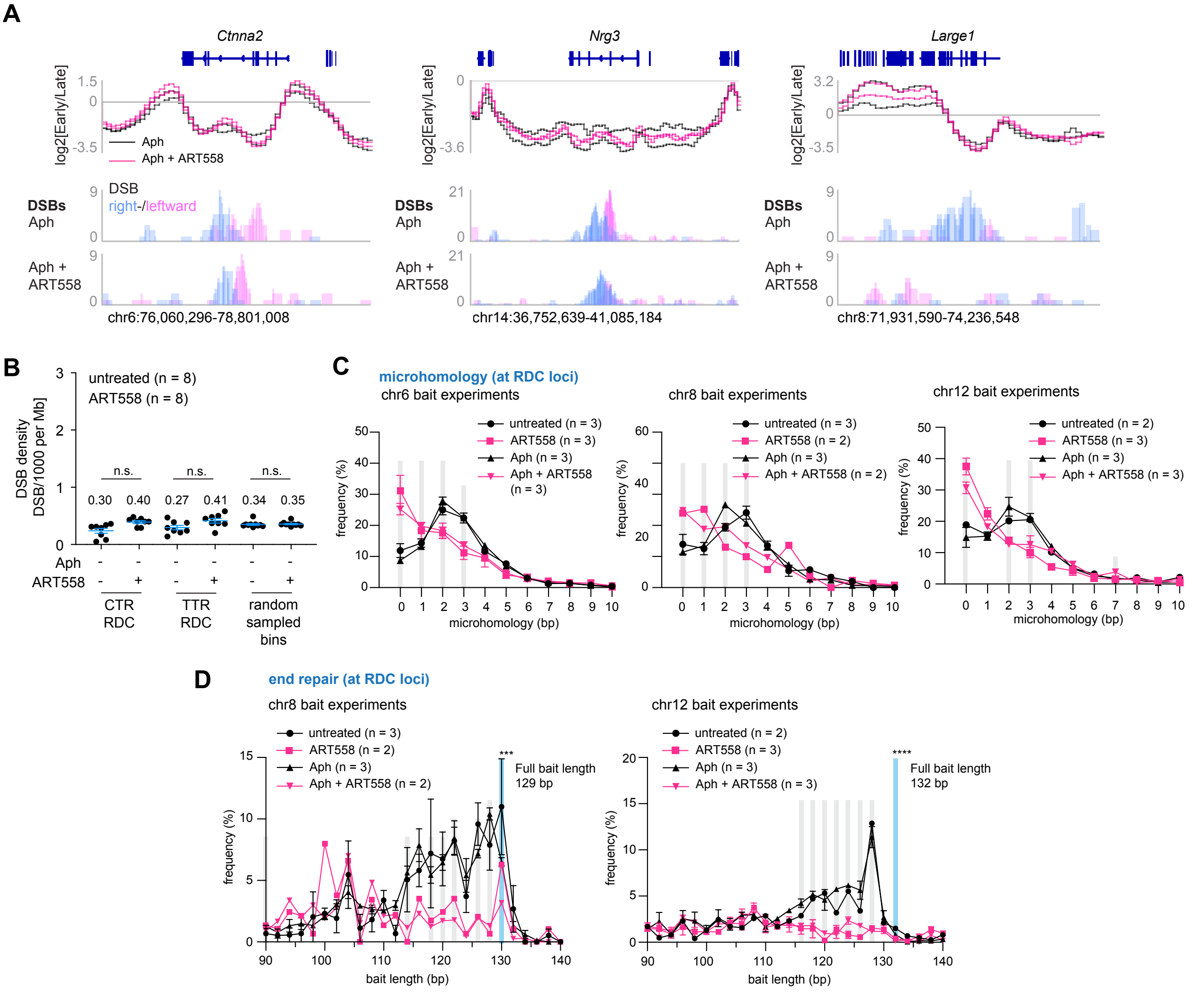


**Supplementary Figure 6. Pol θ inhibition re-wires the repair of replication-stress-induced DSBs. (A)** Genome browser views of three large genes—*Ctnna2* (chromosome 6), *Nrg3* (chromosome 14), and *Large1* (chromosome 8). Upper traces: replication-timing profiles (Repli-seq, $\log_2$ Early/Late) of cells exposed to low-dose aphidicolin (Aph, black) with or without the ATR kinase inhibitor ART558 (magenta). Lower histograms: LAM-HTGTS maps of DSBs obtained from the same samples (blue, Aph; pink, Aph + ART558). Grey horizontal lines mark the genome-wide mean. (**B)** Quantification of DSB density captured by LAM-HTGTS assays in 1520 randomly sampled equal-size genomic bins. Mean for each contusion was shown. Statistical power was determined by a two-tailed t-test. (**C)** Micro-homology frequency at RDC loci translocation junctions at chromosome 6, 8 ,and 12 baits. Mean and SEM were shown. (**D)** Distribution of recovered bait lengths in RDC-loci reads for chromosome 6 and chromosome 12 experiments. Mean and SEM were shown. The total length of bait without end resection is marked by a light blue column (164 bp for the chr14 bait). Bins showing statistically significant differences between APH and APH + ART558 samples, as determined by a two-tailed t-test, are indicated by grey shading. For **(C)** and **(D),** values for individual repeats are shown in Supplementary Tables 2-3; junction numbers analysed per experiment, and statistical significance per bin are shown in Supplementary Table 4. The number of independent biological replicates for each condition is indicated in the label annotation. Source data are provided as a Source Data file.

**Supplementary Figure 7**

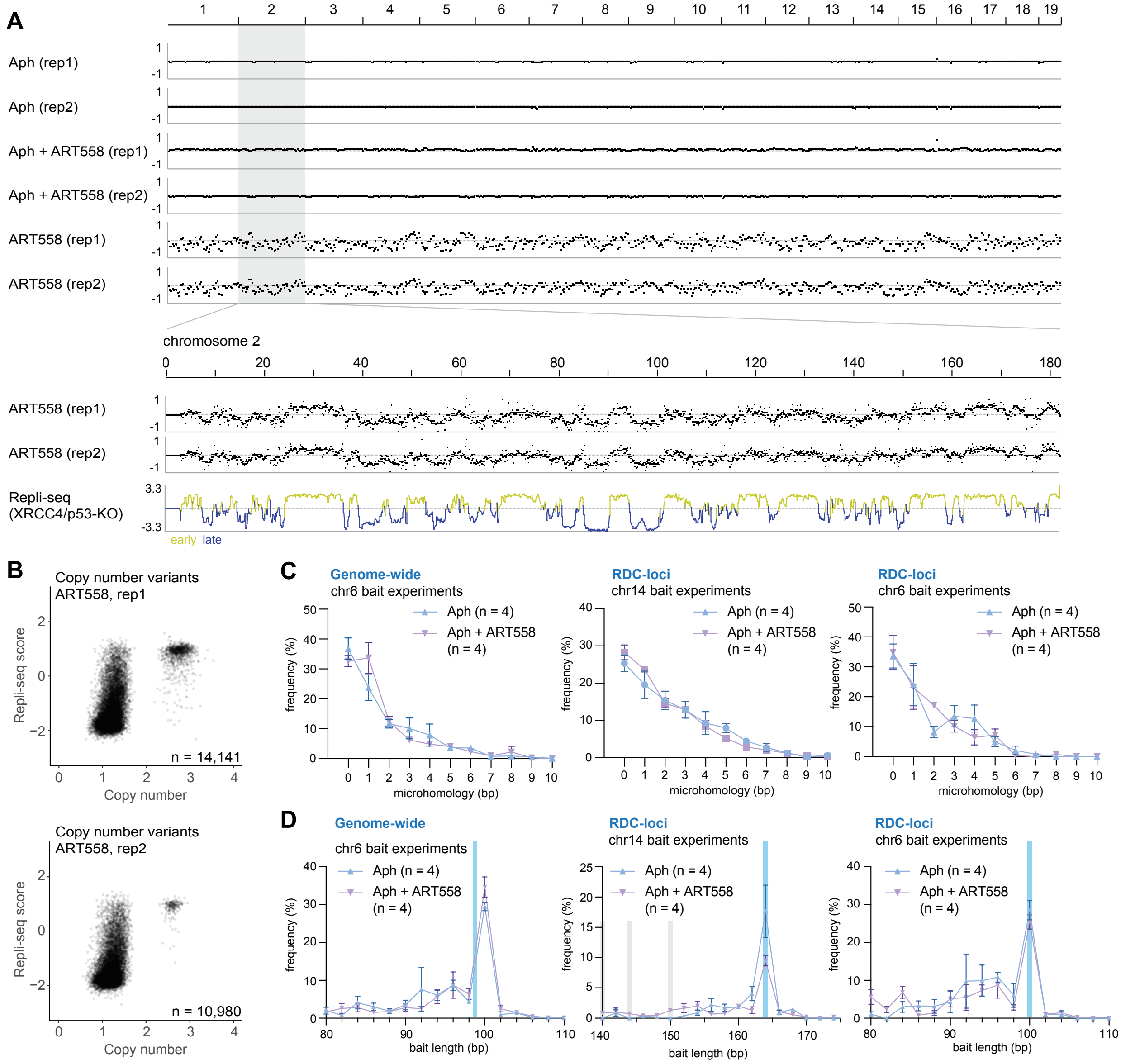


**Supplementary Figure 7. Pol θ inhibition stalled DNA replication, while slightly enhancing direct joining in wild-type neural stem/progenitor cells. (A)** Top panels: Whole-genome sequencing coverage ratios for all autosomes (chromosomes 1–19) across treatment conditions and experimental replicates. Binned sequencing coverage under each condition was normalized to the untreated control (DMSO) and plotted as $\log_2$ ratios (y-axis). Bottom panels: Genome browser view of chromosome 2, showing log2 ratio of genome coverage in cells treated with ART558 alone, and a log2 (early/late) Repli-seq value derived from untreated XRCC4/p53-deficient neural progenitor cells. **(B)** Scatter plots depicting the relationship between copy number and replication timing for significant CNVs detected in two independent ART558-only treatment replicates. The number of CNVs analyzed in each plot is indicated. Kendall's rank correlation, tau = 0.33 for rep1 and 0.27 for rep 2, both has P values smaller than $2.2 \times 10^{-16}$. **(C)** Micro-homology frequency at genome-wide translocation junctions at chromosome 6 baits, and junctions at RDC-loci for chromosome 6 and 14 baits. The figures show mean (dots) and SEM (error bars). Bins showing significant differences between two treatments are highlighted in grey. Statistical significance was determined using a two-tailed t-test. **(D)** Distribution of recovered bait lengths in deletion-sector reads for

chromosome 6 and junctions at RDC-loci for chromosome 6 and 14 baits. An overview of bins 80 - 110 bp (chr6) and 140 - 170 bp (chr14) were shown. The figures show the mean (dots) and SEM (error bars). The total length of bait without end resection is marked by a light blue column (100 bp for the chr6 bait). Statistical significance was determined using a two-tailed t-test, and bins showing significant changes are highlighted in grey. Values for individual repeats are shown in Supplementary Tables 2-3; junction numbers analysed per experiment, and statistical significance per bin are shown in Supplementary Table 4. The number of independent biological replicates for each condition is indicated in the label annotation. Source data are provided as a Source Data file.

## Supplementary Figure 8

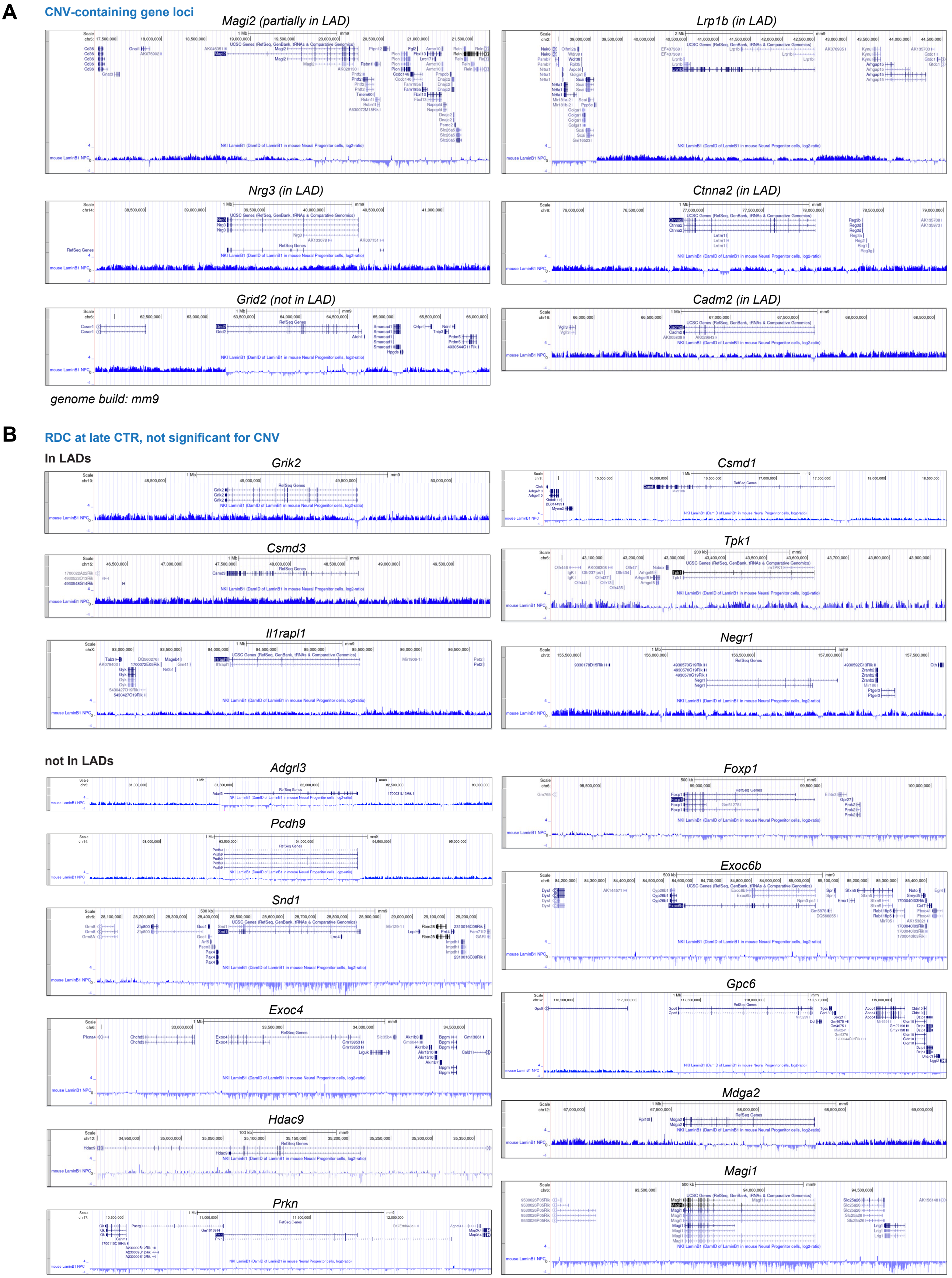


**Supplementary Figure 8. Most CNVs are embedded in the Lamine associated domain (LAD). (A)** Genomic view of CNV loci located either within (*Magi2, Nrg3, Lrp1b, Ctnna2, Cadm2*) or outside (Grid2)

LADs. The UCSC Genome Browser tracks show annotated genes and CNV-containing regions. Lamin B1 DamID signals in mouse neural progenitor cells are plotted as log2 ratios, with positive values indicating lamina association. **(B)** Genomic view of 16 representative late-replicating RDC-containing loci within (upper) or outside (lower) of LADs. Panels are shown as in (A). Lamin B1 DamID data were generated using an array-based approach and mapped to the mm9 genome assembly.
