# Supplementary material for: Recurrent DNA break clusters drive replication-stress-induced copy number variants and genome diversification
Source: Nat Commun. 2026 Apr 20;17:3627. doi: 10.1038/s41467-026-71790-5 (PMC13096501; doi:10.1038/s41467-026-71790-5)
Supplement: Supplementary file 2 — Description of Additional Supplementary Files [file 41467_2026_71790_MOESM2_ESM.pdf]

**Description of Additional Supplementary Files**

**File name:** Supplementary Data 1.

**Description:** This table provides the coordinates and copy numbers for significant CNV loci shown for Figs.1, 4, 5, and 7. CHROM: chromosome, START: start of CNV, END: end of CNV, Copy Number APH: copy number in given CNV locus in cells treated with aphidicolin, ART558: copy number in given CNV locus in cells treated with ART558, or Aph+ART558: copy number in given CNV locus in cells treated with both agents. Gene: Gene overlap with CNV. pNPC: primary wild-type neural stem/progenitor cells. XP-KO: Xrcc4/p53-deficient neural progenitor cells.

**File name:** Supplementary Data 2.

**Description:** This table provides the copy number values shown for the Strand-seq results in Fig. 3. It contains the individual cells displaced in Fig. 3B and D, as well as breakpoints position related to the main result text.

**File name:** Supplementary Data 3.

**Description:** This table summarizes the frequency of microhomology (MH) usage reported in Figures 6, 7, S6 and S7. It presents joining junctions recovered either from genome-wide translocations or from translocations specifically targeting RDC loci. "Chr" denotes the chromosomal bait used in each experiment. MH (bp): microhomology in base-pairs, APH: aphidicolin, ART558: Pol θ inhibitor, and untreated: cells without APH of ART558 treatment.

**File name:** Supplementary Data 4.

**Description:** This table summarizes the frequency of bait length (bp) for reads involving end-joining reported in Figures 6, 7, S6 and S7. It presents joining junctions recovered either from genome-wide translocations or from translocations specifically targeting RDC loci. "Chr" denotes the chromosomal bait used in each experiment. Blen (bp): bait length in base-pairs, APH: aphidicolin, ART558: Pol θ inhibitor, and untreated: cells without APH of ART558 treatment.

**File name**: Supplementary Data 5.

**Description**: This table provides numbers of junctions analyzed for Figs. 6 and 7 experiments. It also contains statistical significance values for results shown for Figs. 6 and 7.

**File name:** Supplementary Data 6.

**Description:** This table provides information for reagents, oligonucleotides, and equipment used for this article. Vender, catalog number, and purposes of assay for each reagent were shown. All reagents are commercially available.
