# Supplementary material for: Recurrent DNA break clusters drive replication-stress-induced copy number variants and genome diversification
Source: Nat Commun. 2026 Apr 20;17:3627. doi: 10.1038/s41467-026-71790-5 (PMC13096501; doi:10.1038/s41467-026-71790-5)
Supplement: Supplementary file 9 — Reporting Summary [file 41467_2026_71790_MOESM9_ESM.pdf]

nature portfolio

Corresponding author(s): Pei-Chi Wei

Last updated by author(s): 03/03/2026

# Reporting Summary

Nature Portfolio wishes to improve the reproducibility of the work that we publish. This form provides structure for consistency and transparency in reporting. For further information on Nature Portfolio policies, see our Editorial Policies and the Editorial Policy Checklist.

## Statistics

For all statistical analyses, confirm that the following items are present in the figure legend, table legend, main text, or Methods section.

| n/a | Confirmed | |
|---|---|---|
| ☐ | ☒ | The exact sample size (*n*) for each experimental group/condition, given as a discrete number and unit of measurement |
| ☐ | ☒ | A statement on whether measurements were taken from distinct samples or whether the same sample was measured repeatedly |
| ☐ | ☒ | The statistical test(s) used AND whether they are one- or two-sided<br>*Only common tests should be described solely by name; describe more complex techniques in the Methods section.* |
| ☒ | ☐ | A description of all covariates tested |
| ☒ | ☐ | A description of any assumptions or corrections, such as tests of normality and adjustment for multiple comparisons |
| ☐ | ☒ | A full description of the statistical parameters including central tendency (e.g. means) or other basic estimates (e.g. regression coefficient) AND variation (e.g. standard deviation) or associated estimates of uncertainty (e.g. confidence intervals) |
| ☐ | ☒ | For null hypothesis testing, the test statistic (e.g. *F*, *t*, *r*) with confidence intervals, effect sizes, degrees of freedom and *P* value noted<br>*Give P values as exact values whenever suitable.* |
| ☒ | ☐ | For Bayesian analysis, information on the choice of priors and Markov chain Monte Carlo settings |
| ☒ | ☐ | For hierarchical and complex designs, identification of the appropriate level for tests and full reporting of outcomes |
| ☒ | ☐ | Estimates of effect sizes (e.g. Cohen's *d*, Pearson's *r*), indicating how they were calculated |

*Our web collection on statistics for biologists contains articles on many of the points above.*

## Software and code

Policy information about availability of computer code

| | |
|---|---|
| Data collection | No software was used. |
| Data analysis | Processing pipelines are available on GitHub as cited in the manuscript: https://github.com/brainbreaks/. |

For manuscripts utilizing custom algorithms or software that are central to the research but not yet described in published literature, software must be made available to editors and reviewers. We strongly encourage code deposition in a community repository (e.g. GitHub). See the Nature Portfolio guidelines for submitting code & software for further information.

## Data

Policy information about availability of data

All manuscripts must include a data availability statement. This statement should provide the following information, where applicable:

- Accession codes, unique identifiers, or web links for publicly available datasets
- A description of any restrictions on data availability
- For clinical datasets or third party data, please ensure that the statement adheres to our policy

Data and code availability: WGS raw reads are deposited in the European Genome phenome Archive under accession PRJEB95922; LAM HTGTS and Repli seq datasets are deposited in GEO (GSE305347), and GRO seq in GEO (GSE305346). Raw Strand-seq data were deposited under EGA (PRJEB105360).

## Research involving human participants, their data, or biological material

Policy information about studies with human participants or human data. See also policy information about sex, gender (identity/presentation), and sexual orientation and race, ethnicity and racism.

| | |
|---|---|
| Reporting on sex and gender | Not applicable. |
| Reporting on race, ethnicity, or other socially relevant groupings | Not applicable. |
| Population characteristics | Not applicable. |
| Recruitment | Not applicable. |
| Ethics oversight | Not applicable. |

Note that full information on the approval of the study protocol must also be provided in the manuscript.

# Field-specific reporting

Please select the one below that is the best fit for your research. If you are not sure, read the appropriate sections before making your selection.

☒ Life sciences ☐ Behavioural & social sciences ☐ Ecological, evolutionary & environmental sciences

For a reference copy of the document with all sections, see nature.com/documents/nr-reporting-summary-flat.pdf

# Life sciences study design

All studies must disclose on these points even when the disclosure is negative.

| | |
|---|---|
| Sample size | Two technical repeats were performed for whole genome sequencing of the parental cell lines, and two fraction Repli-seq. For LAM-HTGTS, three chromosomal baits (chr6, chr8, chr12) were used for Xrcc4/p53-deficient neural progenitor cells. We performed at least two technical repeats for each bait, per condition. For wild-type neural stem and progenitor cells, two chromosomal baits (chr6 and chr14) were used. We performed at least three biological repeats per bait per treatment condition.<br>Strand-seq experiments: No statistical methods were used to pre-determine sample size. Sample sizes were chosen based on technical feasibility and resource constraints to maximize the detection of rare structural variants, consistent with or exceeding standards in the field for single-cell DNA template strand sequencing. We generated a total of 167 high-quality single-cell libraries for analysis: n = 79 cells from the DMSO group and n = 88 cells from the Aphidicolin (Aph)-treated group. This sample size was sufficient to detect recurrent copy number losses and large-scale structural variants. |
| Data exclusions | LAM-HTGTS: For calculating DNA break densities, only DSB detected at the non-bait chromosome were subjected to statistical analyses and plotting. We excluded viewpoint-chromosome for analyses as the DSB recovery rate is biased enriched due to proximity. For calculating microhomology usage, only reads containing none (zero) or 1-10 microhomologies and reads outside of breaksite (+/- 1Mb) were considered for frequency calculation. For bait-length, reads outside of breaksite (+/- 1Mb) were considered for frequency calculation. For ploting Figure 3, reads on the bait chromosomes were used as the gene-of-interests locate on the bait chromosome.<br>For Strand-Seq, we excluded low quality single-cell libraries that showed very low or uneven coverage, or an excess of 'background reads' yielding noisy Strand-seq data prior to analysis. |
| Replication | Data is derived from at least 2 technical repeats, all repeats were successful and where possible data used to perform statistical analysis. |
| Randomization | Depending on cell treatments as described, samples were assigned to corresponding groups in the manuscript. |
| Blinding | Blinding was not performed. All primary outcomes were based on sequencing data analyzed using standardized, automated computational pipelines with predefined parameters, minimizing subjective bias. No experiments involved subjective scoring or behavioral assessment requiring blinding. |

# Reporting for specific materials, systems and methods

We require information from authors about some types of materials, experimental systems and methods used in many studies. Here, indicate whether each material, system or method listed is relevant to your study. If you are not sure if a list item applies to your research, read the appropriate section before selecting a response.

## Materials & experimental systems

| n/a | Involved in the study | |
|---|---|---|
| ☐ | ☒ | Antibodies |
| ☐ | ☒ | Eukaryotic cell lines |
| ☒ | ☐ | Palaeontology and archaeology |
| ☐ | ☒ | Animals and other organisms |
| ☐ | ☐ | Clinical data |
| ☒ | ☐ | Dual use research of concern |
| ☒ | ☐ | Plants |

## Methods

| n/a | Involved in the study | |
|---|---|---|
| ☒ | ☐ | ChIP-seq |
| ☒ | ☐ | Flow cytometry |
| ☒ | ☐ | MRI-based neuroimaging |

# Antibodies

| | |
|---|---|
| Antibodies used | We used anti-BrdU antibody to enrich nascent DNA and RNA. The antibody was purchased from Santa Cruz biotech, cat. no. sc-32323-ac. |
| Validation | This antibody is commercially available. Validation for the assay is provided online on the manufactures' websites. In addition, this antibody was used for a publication by Corazzi et al. to enrich nascent RNA and DNA (https://www.nature.com/articles/s41467-024-47934-w). |

# Eukaryotic cell lines

Policy information about cell lines and Sex and Gender in Research

| | |
|---|---|
| Cell line source(s) | The original mouse Xrcc4/p53-deficient ES cells were described in Tena et al. (https://doi.org/10.1073/pnas.1922299117). |
| Authentication | For derivatives of the original cells generated during this study the genotype was verified by PCR amplification. |
| Mycoplasma contamination | All cell lines were tested for Mycoplasma using the EZ-PCR Mycoplasma testing kit (Biological Industries). Negative results were confirmed by including a +(ve) control provided by the kit. |
| Commonly misidentified lines (See ICLAC register) | No commonly misidentified lines were used in this manuscript. |

# Animals and other research organisms

Policy information about studies involving animals; ARRIVE guidelines recommended for reporting animal research, and Sex and Gender in Research

| | |
|---|---|
| Laboratory animals | Mus musculus (C57BL/6) |
| Wild animals | not applicable. |
| Reporting on sex | both sexes were used |
| Field-collected samples | not applicable. |
| Ethics oversight | We retrieved wild-type embryos from the pregnant female at E17.5. All animal work was performed under institutional license [DKFZ381]. |

Note that full information on the approval of the study protocol must also be provided in the manuscript.

# Clinical data

Policy information about clinical studies

All manuscripts should comply with the ICMJE guidelines for publication of clinical research and a completed CONSORT checklist must be included with all submissions.

| | |
|---|---|
| Clinical trial registration | Not applicable. |
| Study protocol | Not applicable. |
| Data collection | Not applicable. |
| Outcomes | Not applicable. |

## Plants

| | |
|---|---|
| Seed stocks | Not applicable. |
| Novel plant genotypes | Not applicable. |
| Authentication | Not applicable. |
