# Supplementary figures and images for: Recurrent DNA break clusters drive replication-stress-induced copy number variants and genome diversification
Source: Nat Commun. 2026 Apr 20;17:3627. doi: 10.1038/s41467-026-71790-5 (PMC13096501; doi:10.1038/s41467-026-71790-5)

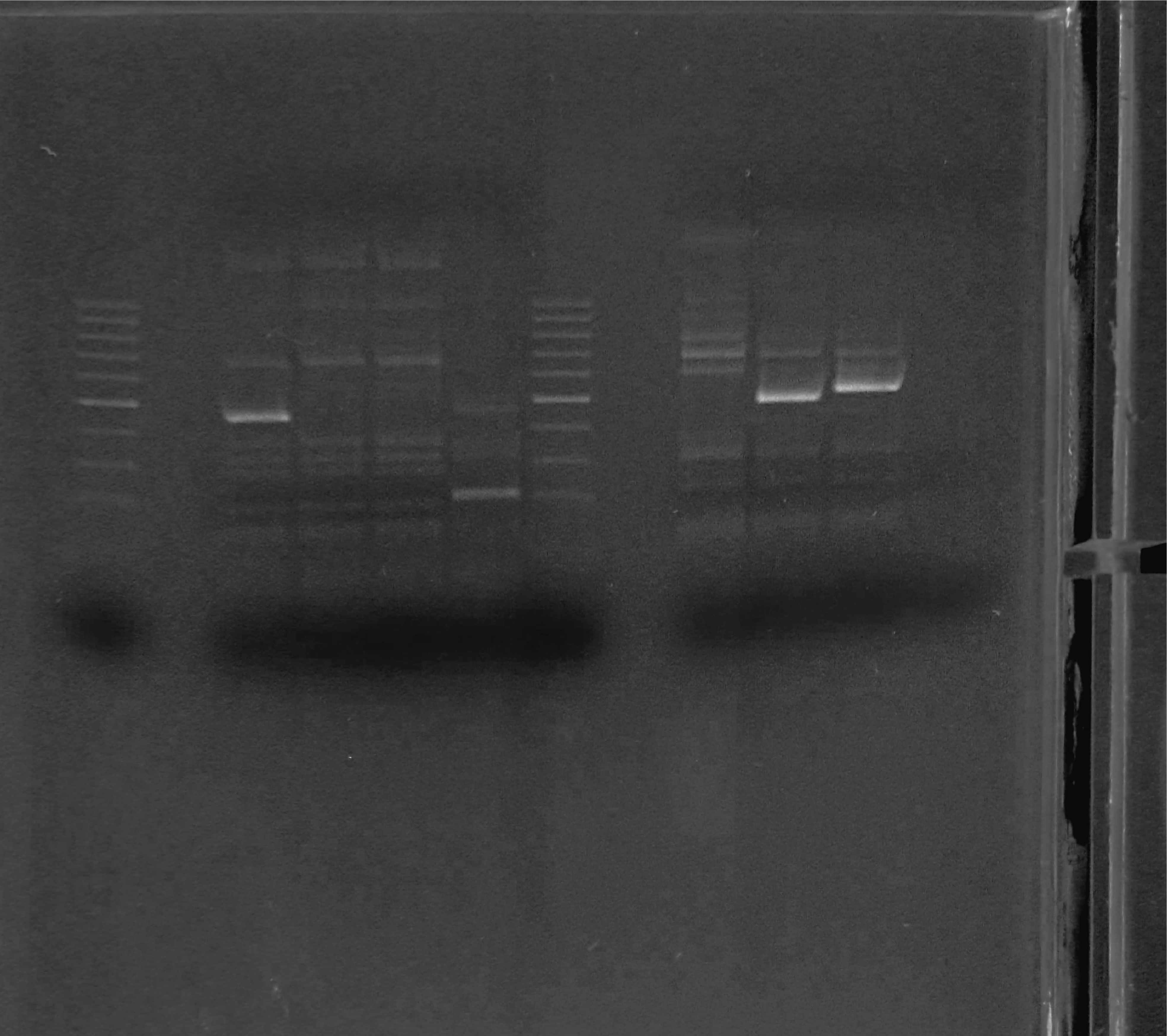

Supplement: Supplementary file 11 — Source Data [file 41467_2026_71790_MOESM11_ESM.zip › Source_data-raw_image_S4.png]
